# Assessing Changes in Surgical Site Infections and Antibiotic Use among Caesarean Section and Herniorrhaphy Patients at a Regional Hospital in Sierra Leone Following Operational Research in 2021

**DOI:** 10.3390/tropicalmed8080385

**Published:** 2023-07-27

**Authors:** Satta Sylvia Theresa Kumba Kpagoi, Kadijatu Nabie Kamara, Ronald Carshon-Marsh, Alexandre Delamou, Marcel Manzi, Rugiatu Z. Kamara, Matilda Mattu Moiwo, Matilda Kamara, Zikan Koroma, Sulaiman Lakoh, Bobson Derrick Fofanah, Ibrahim Franklyn Kamara, Alex Bumble John Kanu, Sartie Kenneh, Joseph Sam Kanu, Senesie Margao, Edward Mberu Kamau

**Affiliations:** 1 Ministry of Health and Sanitation, Government of Sierra Leone (SL), Freetown 00232, Sierra Leone; kamarakadijatunabie@gmail.com (K.N.K.); naldoline@yahoo.com (R.C.-M.); zikankoroma@gmail.com (Z.K.); lakoh2009@gmail.com (S.L.); alexkanu19@gmail.com (A.B.J.K.); sartiekenneh@gmail.com (S.K.); samjoekanu@yahoo.com (J.S.K.); smargao3@gmail.com (S.M.); 2 Department of Nursing, School of Community Health Sciences, Bo Campus, Njala University, Bo 00232, Sierra Leone; 3 Dalla Lana School of Public Health, University of Toronto, Toronto, ON M5T 3M7, Canada; 4 Department of Public Health, Gamal Abdel Nasser University of Conakry, Conakry 00224, Guinea; adelamou@gmail.com; 5 Independent Researcher, 5000 Namur, Belgium; m.manzi449@gmail.com; 6 United States Centers for Disease Control and Prevention, Public Health Emergency Operations Center, Freetown 00232, Sierra Leone; rugiatuzkamara@gmail.com; 7 Ministry of Defense, Republic of Sierra Leone Armed Forces, Freetown 00232, Sierra Leone; mmmoiwo@gmail.com; 8 College of Medicine and Allied Health Sciences, University of Sierra Leone, Freetown 00232, Sierra Leone; kamaramatilda9198@gmail.com; 9 World Health Organization Country Office, 21A-B Riverside Drive, Freetown 00232, Sierra Leone; derrickfbob@gmail.com (B.D.F.); ibrahimfkamara@outlook.com (I.F.K.); 10 UNICEF/UNDP/World Bank/WHO Special Programme for Research and Training in Tropical Diseases (TDR) at the World Health Organization (WHO), 1211 Geneva, Switzerland; kamaued@who.int

**Keywords:** surgical site infection, surgical antibiotic prophylaxis, antibiotic use, caesarean section, herniorrhaphy, SORT IT, AMR, Sierra Leone, pre-operative, post-operative

## Abstract

Surgical site infections (SSIs) are a major public health threat to the success of surgery. This study assessed changes in SSIs and use of antibiotics among caesarean section (CS) and herniorrhaphy patients at a regional hospital in Sierra Leone following operational research. This was a comparative before and after study using routine hospital data. The study included all the CS and herniorrhaphy patients who underwent surgery between two time periods. Of the seven recommendations made in the first study, only one concerning improving the hospital’s records and information system was fully implemented. Three were partially implemented and three were not implemented. The study population in both studies showed similar socio-demographic characteristics. The use of postoperative antibiotics for herniorrhaphy in both studies remained the same, although a significant increase was found for both pre- and postoperative antibiotic use in the CS patients, 589/596 (98.8%) in 2023 and 417/599 (69.6%) in 2021 (*p* < 0.001). However, a significant decrease was observed in the overall incidence of SSIs, 22/777 (2.8%) in 2023 and 46/681 (6.7%) in 2021 (*p* < 0.001), and the incidence of SSIs among the CS patients, 15/596 (2.5%) in 2023 and 45/599 (7.5%) in 2021 (*p* < 0.001). The second study highlights the potential value of timely assessment of the implementation of recommendations following operational research.

## 1. Introduction

A surgical site infection (SSI) is an infection that occurs at or near a surgical incision up to 30 days after surgery without an implant or up to 1 year in patients who have received implants [1]. SSIs are a major public health threat to the success of surgery, especially in low-income countries, where their prevalence is higher than that in high-income countries [2]. According to a 2018 report by the World Health Organization (WHO), the incidence of SSIs in low- and middle-income countries was 11% [3], reinforcing the need to support low-income countries to improve surgical outcomes. In sub-Saharan Africa, SSI occurs in approximately 16% (ranging from 6.8% to 26%) of postoperative wounds [4]. A study conducted by Chu and colleagues in 2015 showed that up to 77% of caesarian sections (CSs) are performed on an emergency basis, and these are more likely to be associated with SSIs compared to those done electively [5].

Previous studies in Sierra Leone have reported the considerable burden of SSIs, with significant morbidity and mortality. Di Gennaro et al. reported a 10.9% incidence of SSIs among CS patients [6], Carshon-Marsh et al. showed an incidence of 6.7% of SSIs among CS and herniorrhaphy patients [7], and Lakoh et al. showed an incidence of SSIs of 11.5% in surgical patients [8]. Superimposed on these challenges of high SSI morbidity is the high rate of inappropriate use of surgical antibiotic prophylaxis (SAP) reported in many hospitals in Sierra Leone [9]. 

The WHO Global Action Plan to tackle antimicrobial resistance (AMR) aims to optimize antibiotic use, including SAP [10]. Even the strategic plan to combat AMR in Sierra Leone strongly emphasized the need to optimize the use of antimicrobial agents [11]. Following these guidelines, the WHO advice is to use a single dose of a first-generation cephalosporin (cefazolin) or penicillin within 120 min of making a skin incision as an intraoperative antibiotic in the case of CS. If the procedure lasts longer than anticipated, a second dose of antibiotic may be necessary [10]. However, for clean procedures, such as surgery for elective hernia repair, the use of SAP or postoperative antibiotics may not be required [12].

Following the 2014–2016 Ebola virus disease (EVD) outbreak in West Africa [13], the National Infection Prevention and Control Unit (NIPCU), with assistance from the WHO, established the SSI surveillance system in the Bo Government Hospital (BGH), southern Sierra Leone. After the EVD outbreak, and during the COVID-19 pandemic, the BGH established and strengthened several infection prevention and control (IPC) measures based on the NIPCU guidelines [14]. However, there have been challenges in the supply chain for IPC commodities, with frequent stockouts of essential IPC materials. In addition, overuse of antibiotics in the context of IPC may lead to AMR in the hospital setting [15]. 

Based on these challenges, an operational research (OR) study supported by the WHO Special Programme for Research and Training in Tropical Diseases (TDR) (hereafter referred to as “the first study”) was conducted in 2021 at the BGH with the aim of assessing the incidence of SSIs and antibiotic use in patients undergoing CS and herniorrhaphy [7]. In the first study, while a high compliance rate with the WHO guidelines was achieved for preoperative SAP, most of the postoperative antibiotics were inappropriately prescribed, and this included 85% of CS patients and 100% of herniorrhaphy patients [7]. 

The first study made seven recommendations: (1) establishment of a hospital antimicrobial stewardship program; (2) education of surgeons, obstetricians and surgical community health officers (SACHOs) on the WHO antibiotic treatment guidelines; (3) monitoring and reporting on antibiotic use; (4) improvement in the hospital’s IPC; (5) improvement in the hospital’s records and information system; (6) review and updating of the national antibiotic treatment guidelines; and (7) a laboratory directorate to strengthen laboratory services for culture and sensitivity at the BGH [7]. 

Since then, there has been no formal assessment of whether these recommendations were adopted and implemented, or of whether these contributed in any way to promoting the rational use of antibiotics and a decrease in the occurrence of SSIs at the BGH. There was, therefore, a need to assess the status of implementation of the recommendations from the first study.

The aim of this study (hereafter referred to as “the second study”) was to document and analyze the change in SSIs and use of antibiotics among patients who underwent CS or herniorrhaphy at the BGH in Sierra Leone between two time periods (November 2019–October 2020 and April 2022–March 2023). The hypothesis underpinning the current study was that the incidence of SSIs would decrease and the inappropriate use of antibiotics among CS and herniorrhaphy patients would reduce at the BGH in response to the 2022 recommendations. Specifically, we aimed to: (1) describe the dissemination activities, decisions, and actions taken to reduce SSIs and promote appropriate use of antibiotics on the maternity and surgical wards of the BGH following the first study; (2) compare the demographic and clinical characteristics between the two studies for CS and herniorrhaphy patients; and (3) compare on the maternity and surgical wards and between the two studies: (i) the incidence of SSIs, (ii) the type and proportion of antibiotics used, and (iii) the timing of antibiotic administration among CS and herniorrhaphy patients. 

## 2. Materials and Methods

### 2.1. Study Design

The second study was a comparative before and after study using routinely collected hospital data among all the patients who underwent CS or herniorrhaphy at the BGH in Sierra Leone between April 2022–March 2023, following the first study conducted from November 2019–October 2020, using the methodology of Carshon-Marsh et al. [7] 

The two studies were not designed from the beginning. However, because of the known implication of operational research on policy and practice, it was later agreed by the authors of the first study to assess the impact of decisions and actions taken based on the recommendations provided in there. As part of the SORT IT program approach, the impact of operational research (OR) study recommendations is routinely assessed. Following the publication of the OR study, the recommendations are disseminated to the relevant stakeholders identified as part of the training activities. The impact assessment activities constitute the second study, which seeks to determine the status of the recommendations derived from the first study while identifying the implementation barriers. 

### 2.2. Study Setting

#### 2.2.1. General Setting

Sierra Leone is a country in West Africa that has an estimated population of over 7.5 million according to the 2021 mid-term population and housing census [16]. It is bordered by Guinea to the north and east, the Atlantic Ocean to the west, and Liberia to the south. It has 16 districts divided into 190 chiefdoms. The main challenges for the healthcare system in Sierra Leone are chronic underfunding, a heavy disease burden, and vastly insufficient numbers of skilled healthcare workers [17]. The gross national income (GNI) per capita (current dollar, purchasing power parity (PPP)) was USD 1750 [18], while the gross domestic product (GDP) growth rate was 3.8% in 2022 [19]. In 2018, the GDP share for health expenditure was 8.29%, and in 2019, it was 8.75% [18].

#### 2.2.2. Specific Setting

**Figure 1:**
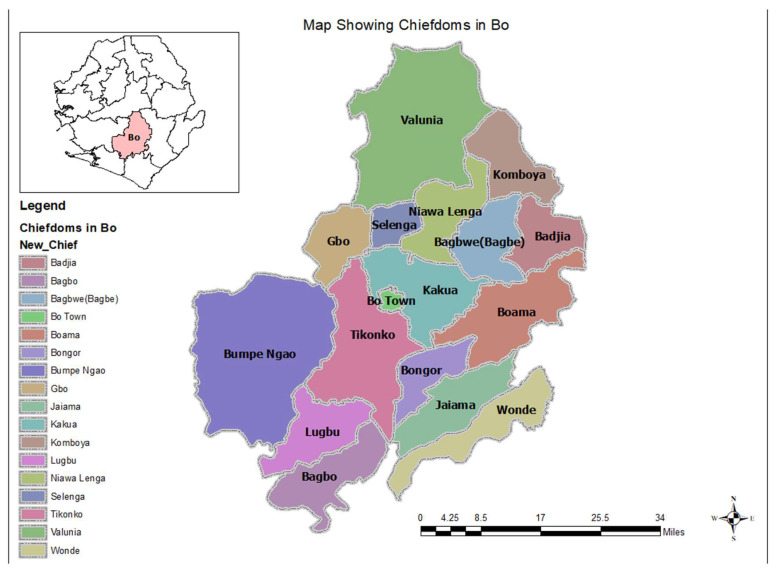
Map of Bo district showing the chiefdoms that form the main catchment areas for the hospital.

Bo district is located in the southern province of Sierra Leone, and it is subdivided into 16 chiefdoms as shown in Figure 1 [20]. The district is the regional headquarters for the southern region. The population based on the 2021 mid-term census was 756,975 [16]. There are 117 health facilities, 1 government hospital, 2 mission hospitals, 4 private hospitals, and 110 peripheral health units (PHUs) (27 community health centers, 21 community health posts and 62 maternal and child health posts).

The second study was conducted at the BGH. It is the main regional referral hospital for the southern province. BGH is located in Bo Town, Kakua Chiefdom. It has 310 beds, 8 doctors (3 at the maternity unit, 3 at the surgical unit, 1 at the neonatal unit and 2 at the outpatient and internal medicine units), 14 surgical community health officers (SACHOs) who assist during surgical procedures and 534 nurses. The hospital has two major theatres, one in the maternity unit and the other called the main surgical theatre. The SSI surveillance system was established in 2019 by the NIPCU with support from the WHO at the maternity unit. The second study data were collected on a paper-based proforma from the theatre registers, dressing room registers and individual patient medical records, and from the principal investigator of the first study, and entered in an EpiData database. Data were entered directly into EpiData (version 4.6.0.6, EpiData Association, Odense, Denmark) by the principal investigator of the second study supported by two data clerks.

### 2.3. Dissemination Details and Recommendations of the First Study

In the second study, the dissemination activities and actions taken based on the recommendations made in the first study were described. The first study was conducted from November 2019 to October 2020. 

The second study was conducted between April 2022 and March 2023. The principal investigator of the second study obtained a descriptive narrative of the dissemination meetings held and the dates, the frequency of use of the dissemination tools and the number and cadre of key personnel who attended the meetings from the first study’s principal investigator. Recommendations and interventions that were proposed by the first study were documented [7]. Routinely collected hospital data (out- and in-patient registers, in- and out-patient charts, theater registers, anaesthetic notes, IPC registers, ward report books, wound dressing books and hospital meeting minute books) were also used to complement this information.

### 2.4. Study Population and Period

Both studies included all the patients who underwent CS or herniorrhaphy during the two study periods. All the patients admitted to the BGH to undergo surgery were enrolled into the SSI surveillance system. Post-discharge surveillance was performed for up to 30 days. After discharge, telephone calls were made by the unit staff to the patients at least twice by the end of the first and second weeks, and the patients normally came for wound dressing and removal of stitches. For those patients referred from distant communities, the staff in charge of the PHU undertook the follow-up of the patients. The patients were referred to the PHU for surgical wound follow-up after discharge and they attended the PHU on scheduled visits during the 30 days post-discharge surveillance period for surgical wound care. The clinicians at the PHU reported any SSIs during the 30-day surveillance period [7]. 

### 2.5. Data Variables

The study data variables are listed according to the study objectives (Table 1).

### 2.6. Data Collection

The first study data were collected in 2021 [7], while the second study data were collected in 2023. To cover the second study objectives, data were collected from the principal investigator of the first study and from routine hospital data on a paper-based proforma extracted from the registers and individual patient medical records. Data were entered directly into EpiData (version 4.6.0.6 EpiData Association, Odense, Denmark) by the principal investigator supported by two data clerks.

In the second study, an SSI was defined as a surgical wound infection that occurred during and up to 30 days of a patient’s admission post-surgery as a result of CS or herniorrhaphy [1]. SSIs were diagnosed based on clinical presentations by the clinician, since culture and sensitivity services were not available at the BGH laboratory. The symptom-based surveillance case definition was adopted from standardized definitions recommended in the WHO protocol for surgical site infection surveillance, with a focus on settings with limited resources [1]. 

### 2.7. Data Validation and Analysis

The data in EpiData were double-entered and validated using EpiData (version 4.6.0.6, EpiData Association, Odense, Denmark). Data analysis was performed using EpiData analysis (v2.2.3.187) software. Descriptive data were summarized using proportions for categorical variables. The timing of antibiotic administration was defined as preoperative (before surgery) and postoperative (after surgery). The proportions of CS and herniorrhaphy patients who received antibiotics as prophylaxis and/or postoperatively were computed. Differences in the proportions of CS and herniorrhaphy patients who received antibiotics as prophylaxis and/or postoperatively between the two periods were assessed using the two-sample z-test for proportions, which is an equivalent of the Chi-square test. A *p*-value < 0.05 was considered statistically significant. The SSI rate was calculated as a percentage of the number of SSI cases recorded over the total number of CS and herniorrhaphy procedures with 30 days of follow-up completed.

## 3. Results

### 3.1. Dissemination Details of the First Study

The principal investigator and team from the first study used various dissemination methods and tools to present their findings and recommendations. These included a plain language handout, short (3 min) and long (10 min) PowerPoint presentations, a published article, and an elevator pitch. A stakeholder mapping was performed to identify key policy and decision makers to whom dissemination should be conducted. The findings and recommendations of the first study were disseminated to the stakeholders identified at different times and places (Table 2).

### 3.2. Recommendations of the First Study

Recommendations were made to the Management Committee of the BGH and to the national AMR committee of the MoHS with the aim of improving the use of postoperative antibiotics and reducing the incidence of SSIs. The status of action is shown in Table 3, and the findings ranged from not implanted to fully implemented.

### 3.3. Socio-Demographic Characteristics of the Study Patients

The second study showed a larger sample size of 777 patients compared to the first study of 681 patients, although both studies were conducted over a 12-month period each. The age group with the highest number of participants was between 15 and 44 years in both studies, 687 (88.4%) for the second study and 642 (94.2%) for the first study. In both studies, most patients lived in urban areas: 546 (70.3%) in the second study and 393 (57.7%) in the first study (Table 4). 

### 3.4. Clinical Characteristics of the Study Patients

The average hospital stay was less than one week in both studies, 649 (83.5%) in the second study and 552 (81%) in the first study. The duration of surgery was also 31–60 min for most of the surgery performed in the two studies, 482 (63.2%) in the second study and 404 (59.3%) in the first study (Table 5).

### 3.5. Surgical Site Infections

Table 6 shows the incidence of SSIs according to the type of surgery. Of the 777 patients in the second study, 596 (76.7%) underwent CS and 181 (23.3) underwent herniorrhaphy compared to 599 (88%) CS and 82 (12%) herniorrhaphy patients in the first study. The total SSI incidence in the second study was 2.8% compared to 6.7% in the first study. The incidence of SSIs in the CS patients was 15 (2.5%) and it was 7 (3.9%) in the herniorrhaphy patients in the second study compared to 45 (7.5%) in the CS patients and 1 (1.2%) herniorrhaphy patient who developed SSIs in the first study. Among the 22 SSI cases in the second study, 15 (68.2%) were CS cases and 7 (31.8%) were herniorrhaphy cases, whereas in the first study, out of the 41 SSI cases, 40 (98%) were in CS patients and only 1 (2%) was from a herniorrhaphy case (Table 6).

### 3.6. Type of Surgical Procedure to the Type of Surgery 

In both studies, most of the surgical operations were emergencies, 486 (65.1%) in the second study and 582 (86.0%) in the first study. Emergency CS accounted for 470 (78.9%) of all the emergency surgery conducted in the second study and 541 (90.3%) in the first study (Table 7). 

### 3.7. Timing and Type of Antibiotics Administered

Table 8 shows the timing of the antibiotic administration, the antibiotics used for pre-operative SAP as well as those prescribed in the postoperative period. The second study found out that 589 (98.8%) of the CS patients and 100% of the herniorrhaphy patients were given antibiotics both preoperatively and postoperatively compared to the first study, where the numbers receiving both preoperative and postoperative antibiotics were 417 (69.6%) for the CS and 58 (70.7%) for the herniorrhaphy patients.

In both studies, intravenous ampicillin and metronidazole were the most common antibiotics used both pre- and postoperatively, while amoxicillin was the most prescribed oral antibiotic postoperatively.

## 4. Discussion

There are a few studies that have assessed and reported on the changes implemented within the health system following operational research recommendations. In the second study, we assessed the changes implemented concerning antibiotic use among patients who underwent CS or herniorrhaphy at the BGH in Sierra Leone between 2021 and 2023. The statuses of the seven recommendations from the first study were assessed as either “not implemented”, “partially implemented” or “fully implemented” based on the actions taken by the respective stakeholders. The results showed that one recommendation was fully implemented, three were partially implemented, and three were not implemented. Factors that might be associated with the recommendations being partially or not implemented may vary from funding issues, the messages not being disseminated to key stakeholders responsible for action and the timing between dissemination and the second study.

A comparison was performed between 681 and 777 surgical patients’ records for the first and second studies, respectively, in the same hospital. The reduction in the sample size in the first study might be due to the fact that elective herniorrhaphies were suspended during the COVID-19 pandemic so as to prevent the spread of COVID-19 amongst healthcare workers. Patients in the second study shared similar socio-demographic and clinical characteristics to those in the first study, except for a different prevalence of co-morbidities.

The overall incidence of SSIs in the second study among patients who underwent CS or herniorrhaphy surgery at the BGH was lower (2.8%) compared to the 6.7% in the first study [7], which is also lower compared to previous studies conducted by Lakoh et al. (11.5%) [8] and Di Gennaro et al. (10.9%) [6]. The reduction in the overall incidence of SSIs could be due to the strengthened IPC measures during the COVID-19 pandemic. This underscores one of the objectives of the WHO and the Sierra Leone national action plan on AMR: That effective infection prevention measures can reduce the incidence of infections [10,11]. This could partly be as a result of partial implementation of the recommendations from the first study [7].

In addition, the incidence of SSIs in the CS patients was also lower (2.5%) compared to 7.5% in the first study, while that of the herniorrhaphy patients was, however, higher (3.9%) compared to 1.2% in the first study [7]. Most of the CS cases in both studies were emergencies, 78.9% in the second study and 90.3% in the first study, whereas 165/181 (91.2%) in the second study and 50% in the first study were elective herniorrhaphy surgical operations. Since emergency surgery is more likely to be associated with SSIs compared to surgery performed electively [5], the lower incidence of SSIs in the second study among the CS cases compared to the herniorrhaphy cases might be associated with the following: The maternity unit undergoing refurbishment, ensuring a larger space for the labour ward and a separate maternity pharmacy. Such infrastructural development has improved IPC compliance. Improved IPC measures and compliance can reduce the incidence of SSIs, as shown by Delamou et al. [21].

A major concern observed during the first study was that most of the postoperative patients were inappropriately prescribed antibiotics, and this included 85% of the CS patients and 100% of the herniorrhaphy patients [7]. In the second study, this situation deteriorated for the CS patients (99.6%) and remained the same for the herniorrhaphy patients at 100%. The WHO SAP guidelines do not recommend the routine use of postoperative antibiotics to reduce the risk of development of AMR [12]. This shows that there is still poor compliance with the WHO SAP guidelines for postoperative antibiotic use at the BGH. The high use of postoperative SAP in the second study might be associated with the fact that surgeons and obstetricians were not trained on the WHO SAP guidelines as recommended by the first study. The intermittent supply of IPC commodities could have resulted in surgeons and obstetricians doubting the effectiveness of the IPC measures in the hospital to adequately prevent SSIs in their patients, as has been reported in a previous study [22]. However, according to De Jonge et al., postoperative SAP has no significant effect on reducing the incidence of SSIs [23].

The commonly used intravenous SAP in the first study, in both surgical procedures, was ampicillin and metronidazole given both preoperatively and postoperatively, with oral amoxicillin given postoperatively. In the second study, however, the type of antibiotic used was the same in the CS cases compared to the first study, but in the herniorrhaphy cases, intravenous ceftriaxone and metronidazole were commonly used. This might be associated with the fact that these antibiotics are provided by the government and are readily accessible [24]. Since culture and sensitivity services are not available at the BGH laboratory, this also hinders the appropriate use of antibiotics, leaving the prescription of the antibiotic to the discretion of the operating surgeon. Other antibiotics prescribed, especially for herniorrhaphies, were oral co-amoxiclav, cefuroxime and azithromycin. It is, however, important to note that azithromycin and cefuroxime are in the WHO watch category, which if not appropriately used, increases the chances of AMR [25].

The second study had the following strengths. First, the study was conducted in a regional referral hospital and the findings are likely representative of patients who underwent CS or herniorrhaphy in this region as well as in many secondary hospitals in Sierra Leone. Second, the current investigator interacted with the previous study team and confirmed with routine hospital data the dissemination activities and status of the recommendations of the first study. Third, data clerks were trained in data collection, and they were supervised by the principal investigator during the two-month study period to ensure data quality. Fourth, the study followed the standard definitions and classifications for eligibility from the WHO SSI surveillance protocol. Finally, the study used the Strengthening the Reporting of Observational Studies in Epidemiology (STROBE) guidelines in its conduct and presentation [26].

The limitations of the second study are the same as those indicted in the first study [7]. Some variables (including timing of surgery, duration of hospital stay, and type of surgical procedure) had missing data. Some variables (including timing of antibiotic administration) would have been useful in assessing compliance, although these data were not collected. Furthermore, we could not make a direct attribution between the first study and the dissemination of its recommendations to the implementation status of the second study. Although the first study’s principal investigator and hospital records provided details of actions taken in response to the recommendations, the reasons why most of the recommendations were not implemented or partially implemented were not provided. There was an overlap between the timing of formal dissemination of first study’s recommendations and the assessment of changes by the second study.

The second study brought out the following operational implications: There is need to implement all the recommendations made by the first study. In addition, further studies are needed to identify the reasons for not implementing or partially implementing most of the recommendations of the first study.

The stakeholders concurred with this structured assessment approach and highlighted its potential role in providing timely feedback that could efficiently identify the strengths and weaknesses of their performance in implementing operational research findings.

## 5. Conclusions

The second study showed a significant decrease in the incidence of SSIs among CS and herniorrhaphy patients at the BGH compared to the first study. The first study showed poor compliance with the WHO SAP guidelines for postoperative surgery, and this situation worsened in the second study. Even though a huge effort was made to disseminate the findings of the first study, most of the recommendations made were not fully acted upon. The recommendations made in the first study are still valid. Additional engagement with the stakeholders is required to improve understanding of the barriers that hindered or the enablers that promoted the implementation of the first study’s recommendations through a qualitative study. This would allow for enhanced routine implementation of operational research findings and scale up of this structured assessment approach in other public health interventions [27].

## Figures and Tables

**Table 1:** Objective-based variables and sources of data of the second study conducted in 2023 on surgical site infections at Bo Government Hospital, Sierra Leone.

Objective	Variables	Sources
Objective 1	Target of dissemination	Previous principal investigator and hospital records
Describe the dissemination activities, decisions, and actions taken to reduce the SSIs and the overuse of antibiotics on the maternity and surgical wards of the * BGH following an operational research study (** SORT IT), led by *** TDR and partners, published in March 2022	Date of dissemination	
	Place of dissemination	
	Mode of dissemination	
	Action taken	
	Date of action	
	Place of action	
Objective 2	Age in years	Wound dressing book, individual patient medical records
To compare the demographic and clinical characteristics between the two study time periods for **** CS and herniorrhaphy patients	Sex (M, F)	
	Residence	
	Referral case or not (cases referred from the ***** PHUs and other hospitals or clinics)	
	Marital status	
	Co-morbidity	
	Date of surgery	
	Diagnosis/indication for surgery	
	Type of surgical procedure	
	Whether elective or emergency surgery	
	The American Society of Anesthesiologists (ASA) score	
	Timing/time of day of operation	
	Duration of the operation	
	Surgical wound classification (four classes from clean to infected wound)	
	Date of admission	
	Date of discharge	
Objective 3	Surgical site infection present: Yes or no	Theatre registers, wound dressing book, individual patient medical records, theatre/anesthetist notes
On the maternity and surgical wards of the Bo Government Hospital and between two time periods (2021 and 2023):	Type of surgical procedure: Caesarean section or herniorrhaphy	
a. To compare the incidence of surgical site infections in CS and herniorrhaphy patients		
b. To compare the type and proportion of antibiotics used among patients.	Antibiotics given: Yes or no	
c. To compare the timing of antibiotics used for CSs and herniorrhaphies.	If antibiotics given: Is it pre- or postoperatively	
	Ampicillin given: Yes or no	
	Gentamycin given: Yes or no	
	Metronidazole given: Yes or no	
	Ceftriaxone given: Yes or no	
	Amoxicillin given: Yes or no	
	Other antibiotics given: Yes or no	

Abbreviations: * BGH—Bo Government Hospital, ** SORT IT—Structured Operational Research Training Initiative *** TDR—For research on diseases of poverty, **** CS—Caesarian section, ***** PHU—Peripheral Health Unit.

**Table 2:** Dissemination details of the findings of the first study conducted in 2021 on surgical site infections at Bo Government Hospital, Sierra Leone.

Mode of Delivery *	To Whom	Where	When
Presentation Published article Social media	Leadership of the MoHS SL	Office of the Chief Medical Officer	April 2022, The CMO is co-author of this article
Presentation Handout/summary brief Published article	Bo Government Hospital management committee	IPC Hall, BGH	May 2022
Presentation Handout/summary brief Published article	Leadership of the National AMR multi-sectorial committee	National SORT IT Dissemination Meeting	May 2022
Clinical meetings Hospital WhatsApp forum	BGH clinicians	IPC Hall, BGH	May 2022

* Dissemination material included a copy of the published article, handout, three minutes of lightening PowerPoint presentation, and ten minutes of technical presentation. Abbreviations: BGH–Bo Government Hospital, IPC—Infection prevention and control, MoHS SL—Ministry of Health and Sanitation Sierra Leone, CMO—Chief medical officer; SORT IT—Structured Operational Research Training Initiative.

**Table 3:** Recommendations from the first study (2021) for improving postoperative antibiotic use safety at the Bo Government Hospital, Sierra Leone, and status of action as of April 2023.

Recommendation	** Status of Action	Details of Action
Hospital antimicrobial stewardship program	Not implemented	At the hospital level, no antimicrobial stewardship program was established. However, at the national level, there were plans to establish hospital-based antimicrobial stewardship programs.
Educate surgeons, obstetricians and surgical * CHOs on WHO antibiotic treatment guidelines	Not implemented	No training on the WHO antibiotic treatment guidelines was provided to surgeons, obstetricians or surgical community health officers.
Monitor and report on antibiotic use	Not implemented	No monitoring or reporting on antibiotic use was performed.
Improve hospital IPC	Partially implemented	Hospital IPC focal points and link personnel conducted daily IPC monitoring and weekly hand hygiene audits. However, the hospital hand sanitizer and liquid soap manufacturing unit was not functional from November 2022, resulting in shortages of supplies.
Improve the hospital’s records and information system	Fully implemented	Child Health and Mortality Prevention Surveillance (CHAMPS) team supported the Bo Government Hospital management in renovating a records room where hard-copy medical files were properly kept.
Review and update the national antibiotic treatment guidelines	Partially implemented	At the national level, funding was sourced, concept notes developed, approval given, and timelines set (second quarter of 2024) for the review of the national standard treatment guidelines and essential medicines list for the inclusion of the *** AWARe classification of antibiotics.
Work with the lab directorate to strengthen laboratory services for specimen culture and sensitivity tests	Partially implemented	Further efforts were made by the laboratory directorate to fast track the restructuring of the three piloted laboratories for specimen culture and sensitivity testing.

* CHOs—Community health officers. ** Status of action; Fully implemented—All actions in line with the recommendation were taken; Partially implemented—Some actions in line with the recommendation were taken; Not implemented—No action taken. *** AWARe—Access, Watch, Reserve.

**Table 4:** Socio-demographic characteristics of patients who underwent caesarean section or herniorrhaphy surgery at the Bo Government Hospital, Sierra Leone, from the first study (2021) compared to the second study (2023).

Characteristics	2021		2023	
	n	* %	n	* %
Total	681	100	777	100
Gender				
Female	599	88	619	79.7
Male	82	12	158	20.3
Age				
<15	1	0.15	0	0
15–44	642	94.5	687	88.4
45–64	26	3.8	72	9.3
≥65	10	1.5	18	2.3
Residence				
Urban	393	57.7	546	70.3
Rural	285	41.8	231	29.7
Referred from peripheral health units				
Yes	212	31.1	207	26.6
No	445	65.4	570	73.4
Not recorded	24	3.52	0	0

* Column percentages.

**Table 5:** Clinical characteristics of patients who underwent caesarean section or herniorrhaphy surgery at the Bo Government Hospital, Sierra Leone, from the first study (2021) compared to the second study (2023).

Clinical Characteristics	2021		2023	
	n	* %	n	* %
Co-morbidity				
Hypertension	33	4.8	101	12.9
Pre-eclampsia	41	6.02	57	7.3
Diabetes mellitus (DM)	0	0	1	0.1
Smoking	2	0.3	1	0.1
Alcoholism	0	0	1	0.8
** Other	2	0.3	59	7.5
Timing of surgery				
Morning	181	26.6	132	17.0
Afternoon	274	40.2	378	48.6
Evening	166	24.4	123	15.8
Night	60	8.8	129	16.6
Not recorded	0	0	15	1.9
Duration of hospital stay				
Less than 1 week	552	89.9	649	83.5
1 week	33	5.4	42	5.4
2 weeks	4	0.6	70	9.0
3 weeks	4	0.6	7	0.9
4 weeks	1	0.2	3	0.4
5 weeks or more	20	3.3	5	0.6
Not recorded	0	0	1	0.1
Duration of surgery				
1 to 30 min	165	24.2	154	20.2
31 to 60 min	404	59.3	482	63.2
61 to 90 min	94	13.8	116	15.2
91 to 120 min	10	1.5	9	1.2
>120 min	7	1.0	0	0
Not recorded	1	0.1	0	0

* Column percentages. ** Other co-morbidities: urinary tract infection, 21 (36%); pelvic inflammatory disease, 15 (25%); syphilis, 11 (19%); benign prostatic hyperplasia, 4 (7%); eclampsia and sickle cell disease, 3 (5%) each; and pulmonary embolism and kidney stone, 1 (2%) each.

**Table 6:** Comparison of the proportions of surgical site infections according to type of surgery at Bo Government Hospital, Sierra Leone, between the first study (2021) and the second study (2023).

Surgeries	2021			2023			** *p* Value
	n	SSI	* %	n	SSI	* %	
Total	681	46	6.7	777	22	2.8	<0.001
Caesarian section	599	45	7.5	596	15	2.5	<0.001
Herniorrhaphy	82	1	1.2	181	7	3.9	0.24

* Percentages calculated out of the total number in each category, ** Two-sample z-test for proportions, SSI = surgical site infections.

**Table 7:** Comparison of the type of surgical procedure to the type of surgery incidence of the surgical site infections among patients who underwent caesarean section or herniorrhaphy at the Bo Government Hospital, Sierra Leone, between the first study (2021) and the second study (2023).

Type of Surgical Procedure	Type of Surgery									
	Caesarean Section					Herniorrhaphy				
	2021		2023		** *p*-Value	2021		2023		** *p* Value
	n	* %	n	* %		n	* %	n	* %	
Elective	58	9.7	95	15.9	0.0012	41	50.0	165	91.2	<0.001
Emergency	541	90.3	470	78.9	<0.001	41	50.0	16	8.8	<0.001
Not Recorded	0	0	31	5.2		0	0	0	0	
Total	599	100	596	100		82	100	181	100	

Tab * Percentages calculated out of the total number in each category, ** Two-sample z-test for proportions.

**Table 8:** Comparison of the choice and timing of antibiotics administered to patients who underwent caesarean section or herniorrhaphy at the Bo Government Hospital, Sierra Leone, between the first study (2021) and the second study (2023).

Choice and Timing of Antibiotics	Type of Surgical Procedure									
	Caesarean Section					Herniorrhaphy				
	2021		2023		** *p*-Value	2021		2023		** *p*-Value
	n	* %	n	* %		n	* %	n	* %	
Patients	599		596			82		181		
Antibiotics										
Ampicillin	549	92	502	84.2	<0.001	57	70	22	12.2	<0.001
Gentamycin	158	26	276	46.3	<0.001	3	4	8	4.4	0.97
Metronidazole	389	65	595	99	<0.001	75	92	181	100	<0.001
Ceftriaxone	121	20	310	52	<0.001	32	39	174	96.1	<0.001
Amoxicillin	552	92	297	49.8	<0.001	42	51	14	7.7	<0.001
Other antibiotics	33	6	45	7.6	0.15	15	18	147	81.2	<0.001
Timing of antibiotics										
Preoperative only antibiotic	88	14.7	2	0.3	<0.001	0	0	0	0	-
Postoperative only antibiotic	94	15.7	5	0.8	<0.001	24	29.3	0	0	-
Both pre and postoperative antibiotics	417	69.6	589	98.8	<0.001	58	70.7	181	100	<0.001

* Percentages calculated out of total number in each category, ** Two sample z-test for proportions.

## Data Availability

Requests to access these data should be sent to the corresponding author.
